# Genomic Landscape of Normal and Breast Cancer Tissues in a Hungarian Pilot Cohort

**DOI:** 10.3390/ijms24108553

**Published:** 2023-05-10

**Authors:** Orsolya Pipek, Donát Alpár, Orsolya Rusz, Csaba Bödör, Zoltán Udvarnoki, Anna Medgyes-Horváth, István Csabai, Zoltán Szállási, Lilla Madaras, Zsuzsanna Kahán, Gábor Cserni, Bence Kővári, Janina Kulka, Anna Mária Tőkés

**Affiliations:** 1 Department of Physics of Complex Systems, Institute of Physics, Eötvös Loránd University, 1117 Budapest, Hungary; 2 HCEMM-SE Molecular Oncohematology Research Group, Department of Pathology and Experimental Cancer Research, Semmelweis University, 1085 Budapest, Hungary; 3 Department of Pathology, Forensic and Insurance Medicine, SE NAP, Brain Metastasis Research Group, Semmelweis University, 1091 Budapest, Hungary; 4 Computational Health Informatics Program (CHIP), Boston Children’s Hospital, Harvard Medical School, Boston, MA 02115, USA; 5 Danish Cancer Society Research Center, 2100 Copenhagen, Denmark; 6 Department of Pathology, Forensic and Insurance Medicine, Semmelweis University, 1091 Budapest, Hungary; 7 Department of Oncotherapy, University of Szeged, 6720 Szeged, Hungary; kahan.zsuzsanna@med.u-szeged.hu; 8 Department of Pathology, Albert Szent-Györgyi Medical Centre, University of Szeged, 6720 Szeged, Hungary; 9 Department of Pathology, Bács-Kiskun County Teaching Hospital, 6000 Kecskemét, Hungary; 10 Department of Pathology, Henry Lee Moffitt Cancer Center and Research Institute, Tampa, FL 33612, USA

**Keywords:** breast cancer, whole-genome sequencing, mutation, germline, somatic

## Abstract

A limited number of studies have focused on the mutational landscape of breast cancer in different ethnic populations within Europe and compared the data with other ethnic groups and databases. We performed whole-genome sequencing of 63 samples from 29 Hungarian breast cancer patients. We validated a subset of the identified variants at the DNA level using the Illumina TruSight Oncology (TSO) 500 assay. Canonical breast-cancer-associated genes with pathogenic germline mutations were *CHEK2* and *ATM*. Nearly all the observed germline mutations were as frequent in the Hungarian breast cancer cohort as in independent European populations. The majority of the detected somatic short variants were single-nucleotide polymorphisms (SNPs), and only 8% and 6% of them were deletions or insertions, respectively. The genes most frequently affected by somatic mutations were *KMT2C* (31%), *MUC4* (34%), *PIK3CA* (18%), and *TP53* (34%). Copy number alterations were most common in the *NBN*, *RAD51C*, *BRIP1*, and *CDH1* genes. For many samples, the somatic mutational landscape was dominated by mutational processes associated with homologous recombination deficiency (HRD). Our study, as the first breast tumor/normal sequencing study in Hungary, revealed several aspects of the significantly mutated genes and mutational signatures, and some of the copy number variations and somatic fusion events. Multiple signs of HRD were detected, highlighting the value of the comprehensive genomic characterization of breast cancer patient populations.

## 1. Introduction

Advanced tools and methods developed in the so-called genomic era have revealed the very complex genetic heterogeneity in breast cancers. Considering the differences across countries in exposure to some of the most important risk factors, such as an unhealthy diet, obesity, consumption of alcohol, sedentary behavior, age at first pregnancy, breastfeeding, and long-term exposure to exogenous hormones, and also in access to genetic counseling and testing, it is difficult to compare the incidence and the relevant mutations in breast cancer emerging in various populations.

Breast cancer still represents a public and a major health concern due to its high morbidity and mortality rates [1,2,3,4]. Although the adjusted breast-cancer-associated death rate is 19.81 per 100,000 of population, making Hungary the fiftieth in the world (based on the latest World Health Organization (WHO) data published in 2020, https://www.wordlifeexpectancy.com/hungary-breast (10 January 2023), the spectrum of breast cancer mutations in Hungarian patients has not yet been deeply analyzed and described.

The frequency and distribution of somatic mutations provide a signature or a map of processes that contribute to tumor development. Comprehensive catalogues of somatic mutations have been developed by large multicenter and multinational projects such as The Cancer Genome Atlas (TCGA) and the International Cancer Genetics Consortium (ICGC) [5,6,7] but are coupled with serious debates regarding implementation in precision or personalized medicine. The growing body of published results related to somatic mutations in breast carcinomas has also produced a wealth of mutational data that allow for the modeling of cancer evolution in greater detail; however, the number and order of events that drive tumor initiation, starting from normal (non-cancerous) cells, remain largely unknown [8]. With the accumulation of sequencing data, the notion of a mutational signature was introduced, reflecting the activity of the mutational processes found to be active throughout a patient’s life. Based on recent studies, more than 30 single-base substitution (SBS) signatures (with only a minority having a verified etiology) were identified [9]. It is relatively well described that the SBS2 and SBS3 signatures present in multiple cancer types are attributed to the enzymatic activity of the APOBEC family of cytidine deaminases and to defects in the DNA damage repair mechanism of HRD, respectively. Other mutational signatures such as SBS4 and SBS7 may reflect various lifestyle factors, such as smoking or UV light exposure, while a few signatures (SBS1, SBS5) are associated with the spontaneous mutational processes of aging [9,10].

Pathogenic variants in the *BRCA1* and *BRCA2* genes are responsible for approximately 20% of hereditary breast cancers [11]. Pathogenic variants detected in other breast cancer susceptibility genes, such as *ATM*, *CHEK2*, *PALB2*, *RAD51*, and *BARD*, may also explain a proportion of the genetic risk [11,12]. This also means that even if germline testing has an impact on therapy in a high percentage of hereditary breast cancers, the underlying genetic contribution to their cancer risk and treatment remains partly unknown [12]. Accordingly, germline predisposition, as well as the reporting of presumed germline pathogenic variants when tumor testing is performed, is still the focus of several studies and debates. Resolving the potential significance of variants previously classified as variants of uncertain significance (VUS) and the identification of additional breast cancer susceptibility genes that could offer clinical utility are important issues. However, a very recent study analyzing germline mutations in BRCA-negative breast cancers concluded that even if additional breast cancer susceptibility genes exist, those of high penetrance are likely to be of very low mutational frequency [13].

Considering the patients’ different lifestyles, and differences in environmental and nutritional factors, it is questionable whether the various genomic findings can be compared across countries. For example, studies comparing Asian breast cancers with tumors from Caucasians show substantial differences in the molecular profiles [14]. Asian breast cancers tend to occur at a younger age, with a higher proportion of estrogen receptor (ER)-negative and human epidermal growth factor receptor 2 (HER2)-positive disease [15].

In Europe, Mathioudaki et al. analyzed the somatic mutational landscape in a Swedish breast cancer cohort. They found that an increased somatic mutation prevalence in the histone modifying genes *KMT2C* and *ARID1A* distinguished the Swedish cohort from patient populations reported in the Catalogue of Somatic Mutations in Cancer (COSMIC) or analyzed in previous studies, and noticed two significantly distinct patterns related to patient age [16]. Another Swedish group, with the aim to improve genetic counseling, have performed exome sequencing on 59 breast cancer (BC) patients from 24 Swedish families with a strong history of BC and have found that several interesting genes, such as the *FANCM* gene, involved in DNA double-strand break repair, and *RAD54L*, were among the mutated genes [17].

Considering the differences in sequencing technologies and in variant calling algorithms, both of which influence the tumor profiling of individual patients over time, researchers will need to collaborate in order to achieve the high patient numbers required to establish large, well-annotated datasets and draw clinically relevant conclusions. The necessity of such efforts is also supported by the fact that breast cancers have become sub-divided into smaller and more defined molecular subgroups and the treatment options are increasingly tending to targeted and personalized therapies that need a deeper understanding of the genomic/molecular background.

Here, we present our attempt (*i*) to characterize the most frequently mutated canonical cancer-related genes in the germline genomes of Hungarian patients with breast cancer; (*ii*) to explore genes most commonly affected by somatic mutations, *(iii)* to analyze the copy number alterations and somatic mutational landscapes of two Hungarian breast cancer cohorts by using whole-genome sequencing (WGS) and the Illumina TruSight Oncology 500 assay, and *(iv)* to compare our results with those observed in various European populations.

## 2. Results

### 2.1. Patient Characteristics

Clinicopathological data for the patients analyzed in the study are listed in Table 1.

### 2.2. Germline Short Variants

#### 2.2.1. Most Frequently Mutated Genes in the Germline Genomes

Germline mutations were investigated in the list of 523 genes contained in the Illumina TruSight Oncology 500 assay (Supplementary Table S2) to allow for straightforward validation of the results. Limiting our analysis of the whole genome in this way also ensures that our results can be interpreted in terms of a cancer-related viewpoint. The analyzed list of genes also included the 21 genes (Supplementary Table S2) listed in the National Comprehensive Cancer Network (NCCN) guidelines for the genetic testing of breast cancer [20]. Variants in either of the “Intron”, “RNA”, “Splice_Region”, “Splice_Site”, “3’UTR”, “5’UTR”, or “Silent” variant classification categories were discarded from the analysis. 

**Figure 1:**
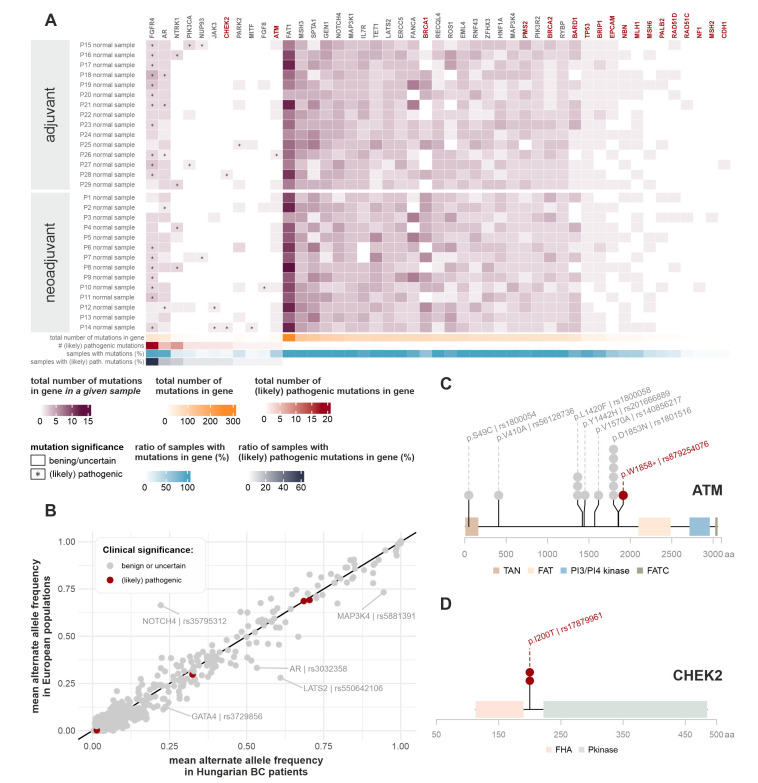
Germline mutations. (**A**) Germline mutations detected in the most frequently mutated and/or (likely) pathogenically mutated TSO genes. Red gene names indicate genes listed in NCCN guidelines for genetic testing of breast cancer. Heatmap colors correspond to the number of germline mutations detected in the given patient, in the given gene. Asterisks mark the presence of (likely) pathogenic germline mutations in the given patient, in the given gene, as annotated by the ClinVar database. Horizontal lower annotations indicate the total number of germline mutations found in the given gene (in all patients), the number of (likely) pathogenic germline mutations found in the given gene (in all patients), the ratio of samples with any type of germline mutation in the given gene, and the ratio of samples with a (likely) pathogenic germline mutation in the given gene, respectively. (**B**) Comparison of observed alternate allele frequencies (AFs) with European cohorts of Genome Aggregation Database (gnomAD) and samples originating from European populations in the 1000 Genomes database. Whenever both databases had available AF data, their mean was used for plotting. If population AF information about the mutation was only present in one of the databases, we used these singular data for the figure. Mutations are colored according to their clinical significance. Outliers are highlighted with the appropriate gene and the RS ID of the given mutation. (**C**,**D**) Germline mutations detected in the *ATM* and *CHEK2* genes. Mutations are indicated with both the consequent amino acid change and their RS ID. Circles above genomic locations display the number of affected samples. Mutations with benign or uncertain significance are marked with grey, and (likely) pathogenic mutations with red colors. Colored blocks along the genes indicate domains present in the Protein Families (PFAM) database [21].

Identified germline mutations in the top 50 most frequently mutated and/or (likely) pathogenically mutated genes are summarized in Figure 1A.

(Likely) pathogenic germline mutations were most frequently identified in the *FGFR4*, *AR*, and *NTRK1* genes. Two patients had a pathogenic germline mutation (rs104886003) in the *PIK3CA* gene. Out of the 21 hereditary breast-cancer-related genes, (likely) pathogenic germline mutations were only present in *CHEK2* (two patients) and *ATM* (one patient) (Figure 1C,D). None of the three affected patients had any recorded family history of cancer.

As an additional, more specific examination of germline mutations, we narrowed our scope to genes listed in De Mattos-Arruda et al. [22] and to the 172 low-risk breast cancer susceptibility genes listed in the recent WHO Breast Tumours volume [23]. Supplementary Figure S1 contains a collection of all germline variants overlapping with these genes. The only (likely) pathogenic mutations in our patient cohort were the ones already discussed above (*PIK3CA*, *CHEK2*, *ATM*). Notably, no *BRCA1/2* germline mutations could be detected in our patient cohorts.

#### 2.2.2. Comparison of Germline Mutation Incidence Rate with Other Patient Cohorts

To assess whether any systematic genomic distinction is present between Hungarian breast cancer patients and generally healthy European populations, the mean alternate allele frequencies of the mutations were compared with those of the European cohort of the 1000 Genomes project and the Genome Aggregation Database (gnomAD) (Figure 1B) [24,25,26]. Outliers are highlighted with the appropriate gene and RS ID of the mutation. The only germline variant associated with inconclusive pathogenic tendencies in the ClinVar database that had a slightly higher frequency in Hungarian patients was rs3729856 in *GATA4*, but given that it has no relation to cancer and its significance status has not been reviewed yet, as of the ClinVar database, it is marked in grey in Figure 1B.

### 2.3. Somatic Mutations

#### 2.3.1. Somatic Tumor Mutational Burden (TMB) and Distribution of Mutational Subtypes

The tumor mutational burden (TMB) is defined as the total number of nonsynonymous mutations per coding area of a tumor genome. The TMB has been identified as a possible biomarker for the response to immune checkpoint inhibitor therapy. We calculated the TMB from the whole-genome sequencing data (Supplementary Figure S2A). Only frame-shift and in-frame deletions and insertions, missense, nonsense, nonstop, splice region, splice site, and translation start site mutations were counted in the analysis. For the adjuvant cohort, the TMB was also determined by the Illumina TruSight Oncology 500 assay as part of the default pipeline. The TMB values fell within the range of 0.5 to 3 mutations/Mbp and 0.8 to 10.2 mutations/Mbp, calculated from the WGS and targeted sequencing data, respectively. The comparison of WGS results with the values obtained from the TSO analysis is presented in Supplementary Figure S2C. The mutational burden is mostly similar for samples collected from the same patient, but varies extensively between different patients, with the only exception for patient P10, for whom the TMB in the core biopsy sample was significantly higher compared to the surgical tissue sample.

We also calculated the somatic mutational prevalence from the WGS data, which is simply the number of somatic mutations identified per Mbp in the genome, disregarding their consequences. It has been previously shown that the somatic mutational prevalence is generally in the range of 0–10 for breast cancer samples, with high variability between patients [27], which is in line with our findings (Supplementary Figure S2B). The criteria for TMB calculation in the TSO panel analysis pipeline are not published in detail and it is possible that the resulting values are better approximations of the somatic mutational prevalence (Supplementary Figure S2D).

Most of the detected somatic short variants in the whole genome were single-nucleotide polymorphisms (SNP) and only around 8% and 6% of them were deletions and insertions, respectively, on average (Supplementary Figure S3B). This is in line with previous literary evidence [28] for most cancer types. Interestingly, when considering only mutations used for TMB calculation, the ratio of insertions becomes substantially lower (Supplementary Figure S3A).

#### 2.3.2. Genes Most Affected by Somatic Short Variants

**Figure 2:**
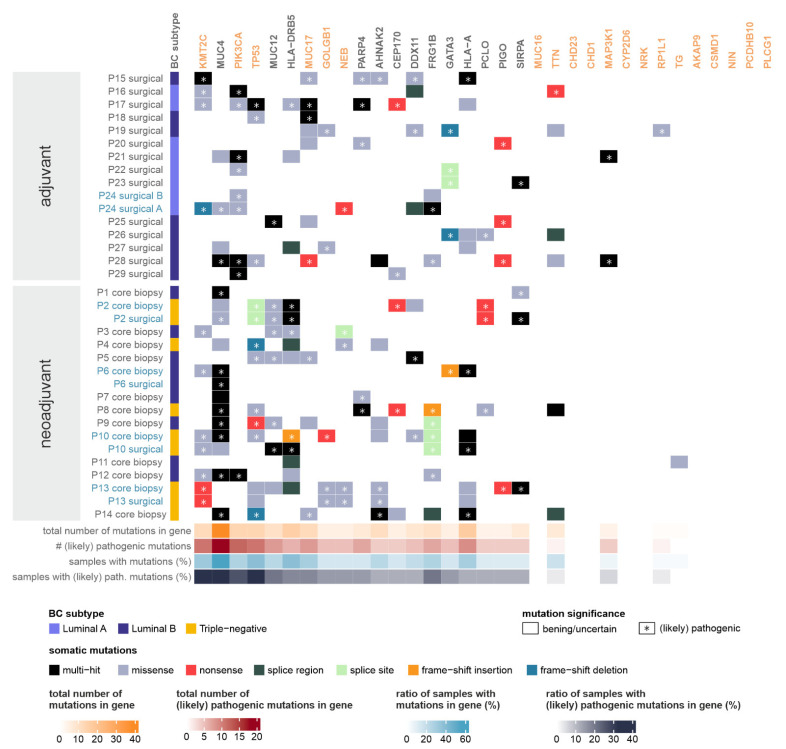
Somatic mutations of moderate or high impact detected in the most frequently mutated and/or pathogenically mutated genes and the genes present in the list of the most mutation-prone genes identified by Mathioudaki et al. in a Swedish BC cohort (marked in orange) [16]. Whenever multiple tumor samples were available from the same patient, the sample names are shown in blue. Vertical left annotation specifies the breast cancer subtype of the tumor. Heatmap colors correspond to the consequence of the somatic mutation detected in the given patient, in the given gene. Asterisks mark the presence of (likely) pathogenic somatic mutations in the given patient, in the given gene, as annotated by the ClinVar database, or deleterious mutations determined by the SIFT or PolyPhen databases. (Whenever multiple somatic mutations are present in a gene (“multi-hit”), an asterisk indicates the presence of at least one (likely) pathogenic mutation.) Horizontal lower annotations indicate the total number of somatic mutations found in the given gene (in all patients), the number of (likely) pathogenic somatic mutations found in the given gene (in all patients), the ratio of samples with any type of somatic mutation in the given gene, and the ratio of samples with a (likely) pathogenic somatic mutation in the given gene, respectively.

The list of genes most prone to somatic mutations, along with the genes identified by Mathioudaki et al. in a Swedish BC cohort [16], are presented in Figure 2. Somatic mutations were filtered to only include those with the subtype of “Frame_Shift_Del”, “Frame_Shift_Ins”, “In_Frame_Del”, “In_Frame_Ins”, “Missense_Mutation”, “Nonsense_Mutation”, “Nonstop_Mutation”, “Splice_Region”, “Splice_Site”, or “Translation_Start_Site”. All mutations that were categorized as (likely) pathogenic by ClinVar, or assumed to be deleterious or damaging by Polymorphism Phenotyping (PolyPhen) [29] or Sorting Intolerant From Tolerant (SIFT) [30], are marked with an asterisk (*). 

The largest number of (likely) pathogenic somatic mutations was found in genes *KMT2C* and *MUC4*, with *PIK3CA* and *TP53* also in the top five genes. *PIK3CA* mutations were exclusively present in tumor samples of luminal subtypes, while *TP53* mutations were significantly more frequent in triple-negative tumors, as expected from previous literary evidence [31,32]. 

Some of the top mutated genes identified in the Swedish BC cohort had similar prevalence in the Hungarian tumor samples (*PIK3CA*—26%, *TTN*—18%), but many of them were mutated more frequently (*TP53*—38%, *KMT2C*—32%, *MUC17*—29%, *NEB*—15%, *GOLGB1*—15%). The rest of the genes (*MUC16*, *CDH23*, *CD1H1*, *MAP3K1*, *CYP2D6*, *NRK*, *RP1L1*, *TG*, *AKAP9*, *CSMD1*, *NIN*, *PCDHB10*, *PLC61*) were typically less likely to be mutated in our cohorts.

### 2.4. Somatic Copy Number Variations

Somatic copy number changes are presented in Supplementary Figure S4 for the most affected genes. The most altered cancer-related genes include *NBN*, *RAD51C*, *BRIP1* (mostly with amplification events), and *CDH1* (usually with losses or deep deletions). It is worth mentioning that in three of the four lobular breast carcinoma cases, *CDH1* loss has been detected. However, it is essential to emphasize that the reliability of the detection of copy number changes profoundly depends on the purity of the tumor samples and also on the purity of the normal samples used as reference. Thus, either a tumor sample with low tumor content or a normal tissue sample somewhat contaminated with tumor cells can cause a significant bias in the estimated copy numbers. To this end, the estimated tumor content of the samples obtained by Sequenza is also presented in Supplementary Figure S4. CNV results for cases with low estimated tumor content should be considered putative and verified with independent approaches, such as microarray-based comparative genomic hybridization (aCGH) or multiplex ligation-dependent probe amplification (MLPA). 

### 2.5. Statistical Patterns of Somatic Mutations

**Figure 3:**
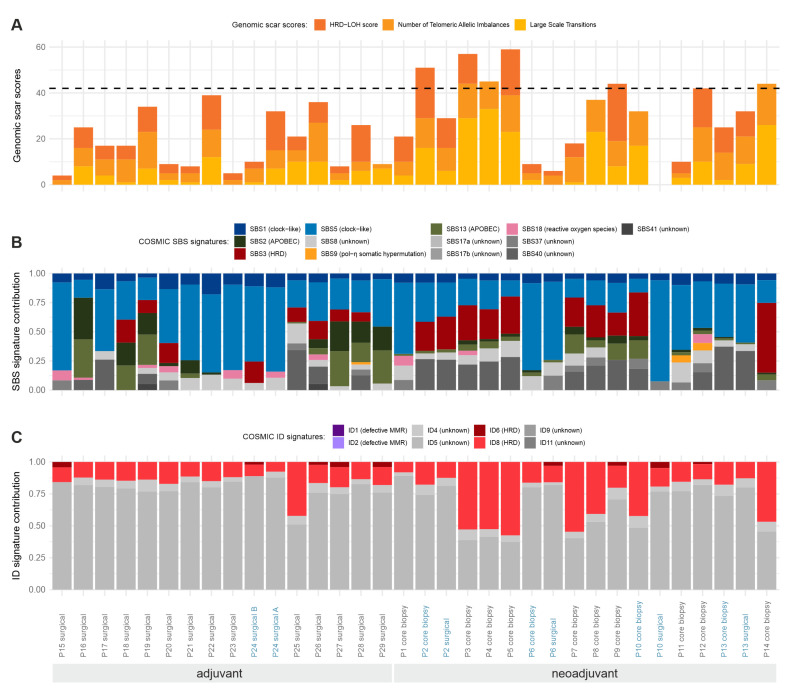
Somatic mutational patterns. Statistical patterns of all somatic mutations identified in WGS data of the investigated samples. Whenever multiple tumor samples were available from the same patient, the sample IDs are shown in blue. (**A**) Genomic scar scores (HRD-LOH score, number of telomeric allele imbalances, and large-scale transitions) and the total HRD score (the sum of these) in the WGS samples determined from the results of the Sequenza tool. The literary threshold [33,34] between “high” and “low” HRD scores is indicated with a horizontal dashed line (HRD = 42). (**B**) COSMIC single base substitution signature contributions to the total somatic SNV load in the samples. Known biological processes connected to the signatures are indicated in the labels. (**C**) COSMIC indel signature contributions to the total somatic indel load in the samples. Known biological processes connected to the signatures are indicated in the labels.

HRD scores of the investigated samples are presented in Figure 3A, along with COSMIC SBS mutational process contributions (Figure 3B) and indel signature decompositions (Figure 3C). Most of the samples fell within the “low” HRD score category (as indicated by the horizontal dashed line in Figure 3A), but, more notably, there were six patients with HRD scores indicating a potential benefit from poly (ADP-ribose) polymerase (PARP) inhibitor therapy. The somatic mutational landscape of the samples was mostly dominated by clock-like signatures (SBS1, SBS5), which had previously been associated with the patients’ age. SBS3, the main process suggesting anomalous homologous recombination in the samples, was also prominent in several investigated samples of the neoadjuvant cohort. On the other hand, the APOBEC-related signatures of SBS2 and SBS15 were usually present in samples of the adjuvant cohort. Similar to SBS3, indel signatures ID6 and ID8, signaling defective homologous recombination, were also present with high proportions in many neoadjuvant samples.

Given that the HRD scores and the proportions of SBS3 and ID6 or ID8 signatures are independent signals of HRD, it was worth investigating whether their values were correlated in the samples (Supplementary Figure S5). As illustrated, the correlations between the pairs of these variables were extremely high; thus, samples identified with their help truly show multiple signs of HRD, even though no pathogenic mutations were found in homologous-recombination-associated genes in any of them.

### 2.6. Somatic Fusion Events and Splice Variants

Gene fusion events and splice variants were determined from the RNA sequencing results of the TSO analysis and are only available for the adjuvant cohort. High-confidence results include the same splice variant in the *AR* gene of the P20 and P21 surgical samples. Reliable fusion events were detected in the P18 surgical sample (*BRCA1/ACSF2*, *RPS6KB1/KIR3DL1; KIR2DS4*, *ZNF254/BRCA1*) and in the P25 surgical sample (*GNAS/KIF5B*). 

### 2.7. Validation of Identified Short Variants

To reveal whether short variants identified by WGS sequencing truly capture the actual mutational events present in samples, we set out to determine whether the mutations listed in the TSO panel results were indeed found by analyzing WGS data and vice versa. Supplementary Figure S6 contains the number of mutations in each detection category for panel sequencing and WGS. When identifying WGS mutations also present in the TSO data, mutations were prefiltered for the TSO gene list (Supplementary Table S2) and also for only exonic mutations, as introns are usually fairly shallowly covered in targeted sequencing setups. 

It is apparent that for germline mutations, the overlap between TSO and WGS results is generally high (>84%). On the other hand, most of the somatic mutations detected by WGS were not found by the TSO panel, and only approximately half of the somatic TSO mutations were deemed reliable by the WGS analysis pipeline. This discrepancy can be explained by the fact that during TSO panel sequencing, only the tumor samples are analyzed, without an appropriate normal control sample. This arrangement requires somatic mutations with low alternate allele frequencies (due to high tumor heterogeneity) to be discarded to avoid the systematic bias introduced by sequencing errors, which could otherwise be compensated by the parallel analysis of normal samples. On the other hand, clonal somatic mutations with high alternate allele frequencies can easily be categorized as germline ones due to the lack of a normal control. Given that, in a strict sense, germline mutations are expected to be present with alternate allele frequencies of either 50% (heterozygous) or 100% (homozygous), their detection is more straightforward; thus, the TSO panel achieves satisfactory results even without the analysis of normal samples. 

## 3. Discussion

Whole-genome (WGS) and whole-exome sequencing (WES) partly revealed the diversity and complexity of somatic and/or germline alterations of breast carcinomas and enabled the discovery of some of the genetic risk variants and acquired somatic mutations driving the disease [12,16,35,36,37,38,39,40,41,42]. Our results contribute to this ever-increasing body of knowledge by describing the genomic peculiarities of breast cancer in a Hungarian patient cohort of homogenous geographic origin.

### 3.1. Germline Mutations

Besides the relatively well-documented hereditary cases explained partly by *BRCA1* and *BRCA2* gene mutations, alterations of several other genes have been identified as susceptibility factors for breast carcinoma, but a significant fraction of the heritability of the disease is still unexplained. Genetic testing for cancer susceptibility genes is still challenging as evidence of an association with cancer is often weak. In our cohort, no *BRCA1/2* pathogenic germline mutations could be detected, whereas (likely) pathogenic germline mutations were present in *CHEK2* (for two patients), *ATM* (for a single patient), and *PIK3CA* (two patients). None of the patients affected by these germline mutations had a family history of cancer, although family history information was limited. By comparing the germline mutation incidence rate with that of other patient cohorts, our results indicate that the observed mutations are approximately as frequent as in independent European populations [36,37,38]. Helgadottir et al., focusing on germline mutations in Swedish breast cancer families, have identified novel breast cancer risk genes such as the *FANCM* gene, involved in DNA double-strand break repair, and the *RAD54L* gene, involved in DNA recombination. They also mention that identifying pathogenic variants is challenging and further studies are needed [17]. Recently published studies assessing the clinical validity of genes frequently tested in hereditary breast and ovarian cancer mostly agree that *ATM*, *BRCA1*, *BRCA2*, *CHEK2*, and *PALB2* are strongly associated with a risk of breast cancer (overall with a *p* value of less than 0.0001) [43,44,45]. Dorling also noticed that four other genes (*BARD1*, *RAD51C*, *RAD51D*, and *TP53*) are associated with breast cancer, with a *p* value of less than 0.05. They also found that approximately 6.8% of the European female patients had protein-truncating variants in any of the nine abovementioned genes, whereas this incidence was 4.4% among Asian women. The difference is partly explained by the significantly lower frequency of the c.1100delC variant in *CHEK2* among Asian women [43].

The CHEK2 and ATM genes are known to be implicated in the development of breast cancer, but the exact association is not clearly understood, and insufficient data are published about the variants classified as having “unknown significance” in these genes [46]. The estimated lifetime risk of acquiring breast cancer with an ATM, CHEK2, PALB2 mutation is greater than 20% which is considered a “moderate risk” compared to the effects of the pathogenic variants in BRCA1 and BRCA2 genes with a lifetime risk of approximately 50%. [47,48,49].

Many of the genes on hereditary breast and ovarian cancer susceptibility panels have not been systematically examined for their associations with the disease and no sufficient evidence has been accumulated for a specific medical management recommendation. More research is needed to determine the magnitude of the cancer risk related to a specific gene alteration and to advise patients who have a variant in the *CHEK2* or *ATM* genes. The presence of *PIK3CA* germline mutations observed in our cohort is an intriguing issue. Dorling and his team used a panel of 34 putative susceptibility genes, including *PIK3CA*, and performed sequencing of 60,466 women with breast cancer and 53,461 control cases, looking for protein-truncating variants and rare missense variants in the 34 genes. There was no evidence of an association between breast cancer and protein-truncating variants in the *PIK3CA* gene [43]. In a very recent study by Kostecka et al., it has been presented that structural chromosomal aberrations and clearly pathogenic point variants in crucial breast cancer driver genes such as *PIK3CA*, *TP53*, *AKT1*, *MAP3K1*, etc., are frequent in the normal mammary glandular tissue that remains after breast-conserving surgery, demonstrating a complex landscape of mutational burden in the seemingly normal mammary glandular tissue [50].

### 3.2. Somatic Mutations

Even if the somatic mutational landscape of breast carcinoma is relatively well documented and the majority of the data agree on the most frequently mutated genes in breast carcinoma, some differences in the prevalence of different mutations are observed. Moreover, some mutations are passenger mutations, and, even among genes known to have driver mutations, variants of unknown significance (VUS) still present a challenge and make it difficult to compare sequencing data generated with various methods. Nik-Zainal et al. found, by analyzing the whole-genome sequences of 560 breast cancer cases, that 93 protein-coding cancer genes carried putative driver mutations. The 10 most frequently mutated genes were *TP53*, *PIK3CA*, *MYC*, *CCND1*, *PTEN*, *ERBB2*, the *ZNF703/FGFR1* locus, *GATA3*, *RB1*, and *MAP3K1*, accounting altogether for 62% of the drivers [7]. 

When comparing our results with the Swedish cohort [16], some of the top mutated genes identified in the Swedish BC cohort had similar prevalence in the Hungarian tumor samples (*PIK3CA*—26%,) but many of them were mutated more frequently (*TP53*—38%, *KMT2C*—32%, *MUC17*—29%, *NEB*—15%, *GOLGB1*—15%). The rest of the genes (*MUC16*, *CDH23*, *CD1H1*, *MAP3K1*, *CYP2D6*, *NRK*, *RP1L1*, *TG*, *AKAP9*, *CSMD1*, *NIN*, *PCDHB10*, *PLC61*) were typically less likely to be mutated in our cohorts. Given that, in the Swedish cohort, except for two cases, only hormone-receptor-positive cases were sequenced, this may explain the observed differences, considering that, in the Hungarian cohort, HR-negative cases were also included. Based on our results, *KMT2C*, *MUC4*, and *TP53* showed the highest mutation counts, followed by *PIK3CA* and *MUC12.* The significance and possible consequences of multiple pathogenic mutations in cancer-related genes within a single sample are generally questionable. 

We observed that there were only two samples that contained mutations in all three of the top mutated genes (*KMT2C*, *MUC4*, and *TP53*) and only one sample had mutations in all three genes with the most mutations in the Swedish cohort (*KMT2*, *PIK3CA*, and *TP53*). The co-occurrence of *PIK3CA* with *KMT2C* mutations was observed in four cases. 

Even though many cancers do not harbor mutations in *MUC* genes, breast cancer appears to be different in this aspect. Compared to the Swedish cohort, where the *MUC17* and *MUC16* genes presented relatively high mutational rates, in the Hungarian cohort, *MUC4* was among the top mutated genes, and mutations in *MUC12* and *MUC17* were detected in seven and six samples, respectively. This is partly explained by the higher number of triple-negative breast cancer (TNBC) cases in the Hungarian cohort, where, in four out of six cases, a mutation was detected in the *MUC4* gene. Chang-Sheng Chang et al. performed mutational profiling of 51 TNBC cases among African Americans (AA) and 77 TNBC cases among Caucasian (CA) patients. Overall, 78% of AA and 90% of CA samples had mutations of the *MUC4* gene. They conclude that *MUC4* remains a molecule of interest in TNBC [51].

### 3.3. Tumor Mutation Burden

The TMB, as the total number of somatic mutations per coding area of a tumor genome, is the focus of several studies as a clinical biomarker associated with the response to immune checkpoint inhibitor (ICI) therapy. The association of a high TMB [52] (“TMB-H”; defined as 10 or more mutations per megabase) with a high response to ICI is documented across different tumor types; however, in breast carcinoma cases, there are several controversies [53,54,55]. A recent study reported that the median TMB was 2.63 mut/Mbp and varied according to the tumor subtype and sample type (metastatic > primary). They found a high TMB in 5% of all breast cancers (more commonly in metastatic tumors), but the prevalence of hypermutated breast cancer is not well described [55]. 

In our study, the TMB values were in the range of 0.5 to 3 mutations/Mbp and 0.8 to 10.2 mutations/Mbp, calculated from the WGS and targeted sequencing data (TSO), respectively. A study by McGrail et al. showed that TMB-H does not predict the response to ICI in all cancer types [56].

Nowadays, the development of treatments that target neoantigens is the focus of several studies [57]. A recent study described a remarkable effect of the adaptive transfer of neoantigen-specific T cells in hormone-receptor-positive metastatic breast cancer patients [47], whereas another study has demonstrated that neoantigens induce anti-tumor immunity in xenograft models [48].

### 3.4. Somatic Mutational Signatures

Recent advancements in cancer therapy make it necessary to not only monitor the list of the most dominantly mutated genes, but to also identify patterns of somatic mutational signatures in the investigated samples. The main challenge in identifying mutational signatures is the several different processes that act over time to generate an individual pattern in each tumor.

The HRD score directly relates to the possible defects of the DNA repair process of homologous recombination. Samples with high HRD scores are likely to have aberrant homologous recombination and thus may be susceptible to PARP inhibitor treatment, even if the classical homologous-recombination-related genes of the sample seem intact. In our samples, where no TMB-H tumors were detected, as in other recently published series [49,58,59], a high frequency of clock-like signatures, processes associated with anomalous homologous recombination, and APOBEC-related signatures were the dominant mutational profiles. In the neoadjuvant cohort, consisting of luminal B1 (LUMB1) and TNBC cases, the SBS3 signature, suggesting anomalous homologous recombination, was more prominent, whereas APOBEC-related signatures (SBS2, SBS13) and signatures associated with the patient’s age (SBS1, SBS5) were more prevalent in the adjuvant cohort. 

Denkert et al. have found that the clinical behavior of a tumor, the response to neoadjuvant chemotherapy, and the disease-free survival of therapy-resistant tumors could be predicted by the mutational signature composition of the tumor. They also found that, in univariate analyses for hormone-receptor-positive tumors, contributions of signatures SBS3 and SBS13 (APOBEC), as well as the exonic mutation rate, were significantly correlated with increased pathological complete response rates. They concluded that mutational signatures might be used in identifying tumors with an increased response rate to neoadjuvant chemotherapy and to define therapy-resistant subgroups for future therapeutic interventions [49].

We refrained from performing statistical analyses between responder and non-responder cases in the neoadjuvant cohort, as 4/14 cases showed pathological complete regression (pCR). Three out of four cases achieving pCR presented SBS3 and SBS13 signatures.

In the Swedish cohort, the most frequent mutational signatures were COSMIC signature 2 (attributed to APOBEC deaminase activity), COSMIC signature 6 (associated with a defective DNA mismatch repair mechanism), and COSMIC signature 5 (a clock-like signature with mostly unknown etiology) [16].

A study analyzing the repertoire of mutational signatures in different cancer types presented that, in breast carcinomas, similarly to our results, the most frequent signatures with proposed etiologies were SBS1 (clock-like, deamination of 5-methylcytosine), SBS2 (APOBEC activity), SBS5 (unknown), and SBS13 (APOBEC activity), followed by SBS3 (defective homologous recombination DNA repair) and SBS18 (reactive oxygen species). They also observed substantial variation between cases regarding the number of indels detected in them. The most frequent ID signatures in our cohort were ID4 and ID5 with unknown function, and the ID6 and ID8 signatures associated with DNA double-strand break repair by non-homologous end joining, which can also be a sign of defective homologous recombination. Based on the data of Alexandrov et al., ID6 and ID8 are characterized predominantly by ≥ 5-bp deletions and the contribution of ID6 tends to correlate with that of SBS3. On the other hand, the presence of ID8 did not correlate strongly with that of SBS3 [9]. In our cohort, SBS3, the main process suggesting anomalous homologous recombination in the samples, is prominent. Indel signatures ID6 and ID8, also signaling defective homologous recombination, are present with high proportions, especially in neoadjuvant samples. Considering the HRD score, most of our samples fell within the “low” HRD score category, but, more notably, there were six patients with HRD scores that could indicate a potential benefit from PARP inhibitor therapy.

### 3.5. Somatic Copy Number Variations

Mathioudaki, A et al. [16] have detected *CDK10*, a known tumor suppressor, to be significantly deleted, and *MDM4*, an oncogene, to be amplified in a large fraction of their samples, although they noted that targeted sequencing is not the optimal method for the identification of large-scale genomic events. Moreover, they have found an amplified segment on chromosome 6 involving genes such as *ABBC10* and *ZNF318.* The majority of the CNVs that have been identified to date for breast cancer are rare in familial breast cancer and are more challenging to detect with current technologies than single-nucleotide variants (SNVs) [56]. We have found that the most altered cancer-related genes include *NBN*, *RAD51C*, *BRIP1* (mostly with amplification events), and *CDH1* (usually with losses or deep deletions).

The interest in exploring the role of fusion genes in the development and progression of breast carcinoma and other types of cancer has significantly increased; however, based on recent studies, most of the detected gene fusions seem to be “passenger” events and the presence of recurrent and driver fusions is still under serious debate [60].

Overall, we detected a few novel gene fusion events in two samples, and could not confirm the previously reported fusions. Further investigation is necessary to comprehend the biological significance of these aberrations.

## 4. Materials and Methods

### 4.1. Patients and DNA Extraction

Our study included the whole-genome sequencing of 63 samples from 29 patients diagnosed with breast carcinoma (ethical approval: 14383-2017 ETT-TUKEB).

The study consisted of two patient cohorts, stratified based on treatment protocol. The adjuvant cohort included 16 primary tumor surgical samples of 15 patients (one with samples from two different localizations) and their 15 matched adjacent normal breast tissue samples. These patients received no chemotherapy prior to surgery. The neoadjuvant cohort consisted of 14 tumor core biopsy samples of 14 patients obtained prior to neoadjuvant chemotherapy, 14 corresponding surgical samples of adjacent normal breast tissue, and 4 additional surgical tumor samples obtained after neoadjuvant treatment.

Samples were collected between 2004 and 2017 in the adjuvant cohort and between 2012 and 2014 in the neoadjuvant cohort, at the Department of Pathology, Forensic and Insurance Medicine, Semmelweis University, Budapest, Hungary and at the Department of Pathology, University of Szeged, Hungary, respectively. Tissue was frozen at −80 °C until DNA extraction. Genomic DNA was extracted with the QIAamp DNA Micro Kit (50), according to the manufacturer’s protocol. DNA quantification was performed with a Qubit fluorometric system (Life Technologies, Waltham, MA, USA).

### 4.2. Whole-Genome Sequencing

Next-generation sequencing libraries were prepared from 1 μg DNA input material using the TruSeq DNA PCR-Free Library Preparation Kit (Illumina) with IDT for Illumina TruSeq UD Indexes (Integrated DNA Technologies, Coralville, IA, USA). Briefly, genomic DNA was sheared using a Covaris S220 focused ultrasonicator instrument (Woburn, MA, USA); DNA fragments were cleaned, end-repaired, and 3’ A-tailed, followed by ligation of the sequencing adapters. After quality control, individual libraries were diluted, equimolarly pooled, and sequenced on an Illumina NovaSeq 6000 instrument (San Diego, CA, USA) using an S4 flow cell and 2 × 150 bp paired-end (PE) chemistry. Library preparation and sequencing was performed in the Biomedical Sequencing Facility at CeMM—Research Center for Molecular Medicine of the Austrian Academy of Sciences (Vienna, Austria).

### 4.3. Bioinformatic Analysis—Short Variant and CNV Detection

The URLs of the accessed websites and the software versions of different tools are listed in Supplementary Table S1.

Quality control of raw sequencing data was performed with the FastQC and multiQC software tools. Unaligned uBAM files were first converted to FASTQ with Picard tools and aligned to the human reference genome (version hg38) with the bwa mem algorithm. Duplicate reads were marked and the Genome Analysis Toolkit (GATK) BQSR pipeline was used to recalibrate the base quality scores. 

Short somatic mutations were detected in tumor–normal sample pairs with Mutect2 and further refined with the FilterMutectCalls GATK tools. Germline short variants were identified in normal adjacent tissue samples with the use of the HaplotypeCaller, GenomicsDBImport, and GenotypeGVCFs GATK tools, and further filtered with the Variant Quality Score Recalibration pipeline. 

Short genomic variants were annotated with the Ensembl Variant Effect Predictor (VEP, v94), using the ClinVar (v201706) [61,62], dbSNP (v150) [62], COSMIC (v81) [63], 1000 Genomes (phase3) [64], and gnomAD (v170228) [26] databases. 

Comparison of the observed aggregated alternate allele frequencies of mutations with available data from other databanks (1000 Genomes, gnomAD) was performed by calculating the ratio of the total number of alternate alleles in mutated samples and the total sequencing depth, while assuming that non-mutated samples contained exactly zero alternate alleles in the given genomic position and had the same sequencing depth as the mean depth of mutated samples.

Somatic variation spectra were decomposed into weighted contributions of COSMIC single base substitution (SBS) and indel (ID) signatures (v3.3) in R (v4.2.1), using an expectation-maximization approach.

Different copy number segments along the whole genome were called with Sequenza (v3.0.0) [65], which also provided estimated tumor content for all investigated samples.

The calculation of the genomics scar scores (loss of heterozygosity (LOH) [66], large-scale transitions (LST) [67], and number of telomeric allelic imbalances (ntAI)) was performed using the scarHRD R package (v0.1.1) [68].

### 4.4. Validation and RNA Variants

RNA was extracted with the RNeasy Micro Kit (50) (Qiagen) according to the manufacturer’s protocol. RNA quantification was performed with a Qubit fluorometric system (Life Technologies).

For the adjuvant cohort, the Illumina TruSight Oncology (TSO, San Diego, California, USA) 500 assay was used to validate a subset of the identified variants at the DNA level in a preselected set of 523 genes. The TSO 500 analysis was performed in the Biomedical Sequencing Facility at CeMM—Research Center for Molecular Medicine of the Austrian Academy of Sciences (Vienna, Austria).

This approach also allowed for the screening of single-nucleotide variants, small insertions and deletions in 151 genes, amplifications in 59 genes, and gene fusions plus splice variants in 55 genes at the RNA level.

## 5. Conclusions

The only (likely) pathogenic germline mutations in our patient cohort occurred in the *PIK3CA*, *CHEK2*, and *ATM* genes. Notably, no *BRCA1/2* germline mutations could be detected, likely due to the limited size of our patient cohort. A high frequency of clock-like signatures, processes associated with anomalous homologous recombination, and APOBEC-related signatures were the dominant mutational profiles. In the neoadjuvant cohort, consisting of LUMB1 and TNBC cases, the SBS3 signature, suggesting defective homologous recombination, was more prominent, whereas APOBEC-related signatures (SBS2, SBS13) and signatures associated with the patient’s age (SBS1, SBS5) were more prevalent in the adjuvant cohort. Even though no pathogenic mutations were found in homologous-recombination-associated genes, multiple signs of HRD were detected, highlighting the value of the comprehensive genomic characterization of BC patient populations with a highly localized geographical distribution.

## Figures and Tables

**Table 1:** Clinicopathological characteristics of the patients. ER, estrogen receptor; PR, progesterone receptor; HER2, human epidermal growth factor receptor 2; SD, standard deviation; NA, data not available.

Variable	Adjuvant Cohort (*n* = 15)	Neoadjuvant Cohort (*n* = 14)	
Age (years)			
Mean ± SD	58.93 ± 13.23	54.71 ± 9.93	
Range	32–80	37–67	
Tumor tissue histological subtypes	16 tumor samples	18 tumor samples	
		core biopsy	surgical
Invasive lobular carcinoma (ILC)	4 (25.0%)	0 (0%)	0 (0%)
Invasive breast carcinoma of no special type (NST)	11 (68.8%)	14 (77.8%)	3 (16.7%)
Mixed ductal and lobular carcinoma	1 (6.2)		
Metaplastic carcinoma	0 (0%)	0 (0%)	1 (5.5)
Pathological tumor size (pT)	pT	ypT	
T0	0 (0%)	4 (28.5%)	
T1	4 (27%)	5 (36%)	
T2	10 (67%)	4 (28.5%)	
T3	1 (6%)	1 (7%)	
Pathological lymph node status (pN)	pN	ypN	
pN0	9 (60%)	7 (50%)	
pN1	3 (20%)	5 (36%)	
pN2	2 (13%)	1 (7%)	
pN3	1 (7%)	1 (7%)	
No	14 (93%)	11 (78%)	
Yes	1 (7%)	2 (14%)	
NA	0 (0%)	1 (7%)	
ER status			
Positive	15 (100%)	8 (57%)	
Negative	0 (0%)	6 (43%)	
PR status			
Positive	11 (73%)	6 (43%)	
Negative	4 (27%)	8 (57%)	
HER2			
Positive	1 (7%)	0 (0%)	
Negative	14 (93%)	14 (100%)	
IHC molecular subtype ^1^			
Luminal A	7 (47%)	0 (0%)	
Luminal B	8 (53%)	8 (57%)	
Triple negative	0 (0%)	6 (43%)	
Tumor response to neoadjuvant therapy ^2^			
TR1a		4 (28.5%)	
TR1b		0	
TR2a		0	
TR2b		5 (36%)	
TR2c		4 (28.5%)	
TR3		0	
NA		1 (7%)	
Lymph node response to neoadjuvant therapy ^2^			
NR1		0	
NR2		1 (7%)	
NR3		4 (28.5%)	
NR4		0	
NA		9 (64.5%)	

^1^ IHC molecular subtype as defined by 2013 St. Gallen Consensus Conference [18]. ^2^ Assessment of degree of response to chemotherapy [19].

## Data Availability

The data presented in this study are available at the EGA database, under accession ID EGAS00001007196 upon the approval of the Data Access Committee affiliated with the dataset.

## Supplementary Materials

The following supporting information can be downloaded at: https://www.mdpi.com/article/10.3390/ijms24108553/s1.

## Abbreviations

ER	Estrogen Receptor
ICGC	International Cancer Genetics Consortium
ID	Indel
GATK	Genome Analysis Toolkit
HER2	Human Epidermal Growth Factor Receptor 2
HRD	Homologous Recombination Deficiency
IDC-NST	Invasive Ductal Carcinoma of No Special Type
ILC	Invasive Lobular Carcinoma
PR	Progesterone Receptor
SNP	Single-Nucleotide Polymorphism
TCGA	The Cancer Genome Atlas
TSO	TruSight Oncology
TMB	Tumor Mutation Burden
VUS	Variant of Uncertain Significance
WGS	Whole-Genome Sequencing
WES	Whole-Exome Sequencing
