# Supplementary material for: Genomic Landscape of Normal and Breast Cancer Tissues in a Hungarian Pilot Cohort
Source: Int J Mol Sci. 2023 May 10;24(10):8553. doi: 10.3390/ijms24108553 (PMC10218458; doi:10.3390/ijms24108553)
Supplement: Supplementary file 1 [file ijms-24-08553-s001.zip › SupplementaryTable1.pdf]

Supplementary Table 1. URLs of the accessed websites and tools

**Tool/resource**	**URL**	**Accessed**
FastQC (v0.11.8)	https://www.bioinformatics.babraham.ac.uk/projects/fastqc	26th September 2022
multiQC (v1.7)	https://multiqc.info	26th September 2022
Picard tools (v2.20.0)	https://broadinstitute.github.io/picard/	26th September 2022
SAMtools (v1.5)	http://samtools.sourceforge.net/	26th September 2022
bwa (v0.7.12-r1039)	http://bio-bwa.sourceforge.net	26th September 2022
GATK (Genome Analysis Toolkit; v4.0.10.1)	https://software.broadinstitute.org/gatk	26th September 2022
BQSR pipeline (Base Quality Score Recalibration)	https://software.broadinstitute.org/gatk/documentation/tooldocs/3.8-0/ org_broadinstitute_gatk_tools_walkers_bqsr_BaseRecalibrator.php	26th September 2022
VEP (Ensembl Variant Effect Predictor; v94)	https://www.ensembl.org/vep	26th September 2022
COSMIC (Catalogue of Somatic Mutations in Cancer; v3.3)	https://cancer.sanger.ac.uk/cosmic/signatures	26th September 2022
