# Supplementary material for: Genomic Landscape of Normal and Breast Cancer Tissues in a Hungarian Pilot Cohort
Source: Int J Mol Sci. 2023 May 10;24(10):8553. doi: 10.3390/ijms24108553 (PMC10218458; doi:10.3390/ijms24108553)

Supplementary Figure 2. Tumor mutational burden (TMB) and somatic mutational prevalence in investigated samples.
Whenever multiple tumor samples were available from the same patient, appropriate bars and sample names are shown in blue. **A.** Tumor mutational burden (TMB) determined from WGS data in the analyzed samples. **B.** Somatic mutational prevalence determined from WGS data in the analyzed samples. **C.** Relationship between TMB calculated from WGS and data and from the Illumina TruSight Oncology 500 assay (TSO). **D.** Relationship between somatic mutational prevalence calculated from WGS and TMB calculated from TSO data.

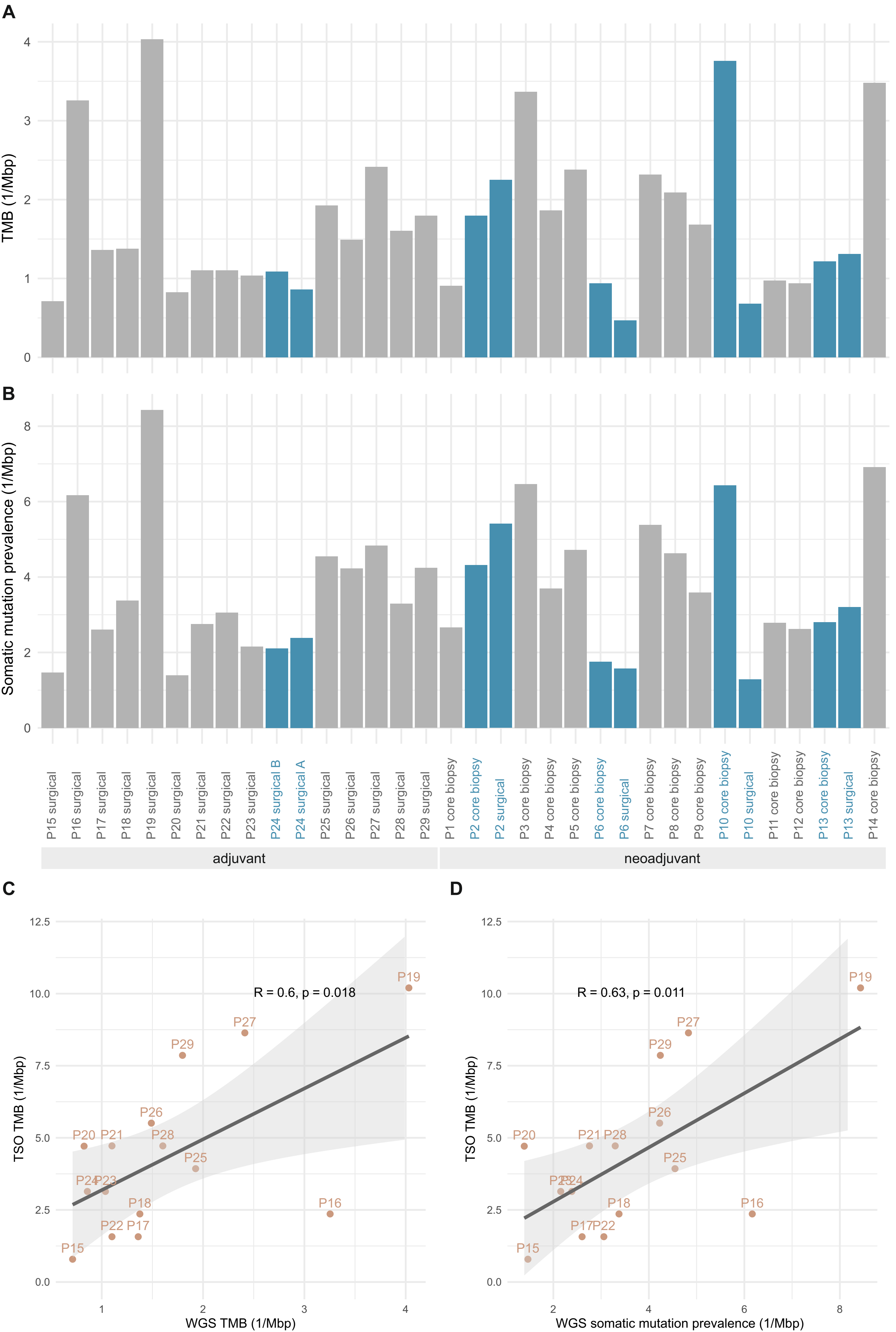

Supplement: Supplementary file 1 [file ijms-24-08553-s001.zip › Supp_Fig_2_new.pdf]
