# Supplementary material for: Genomic Landscape of Normal and Breast Cancer Tissues in a Hungarian Pilot Cohort
Source: Int J Mol Sci. 2023 May 10;24(10):8553. doi: 10.3390/ijms24108553 (PMC10218458; doi:10.3390/ijms24108553)

Supplementary Figure 3. Distribution of mutational subtypes found in the analyzed samples. Whenever multiple tumor samples were available from the same patient, appropriate sample names are shown in blue. **A.** Ratio of mutational subtypes in case of mutations considered for TMB calculation. **B.** Ratio of mutational subtypes for all somatic mutations.

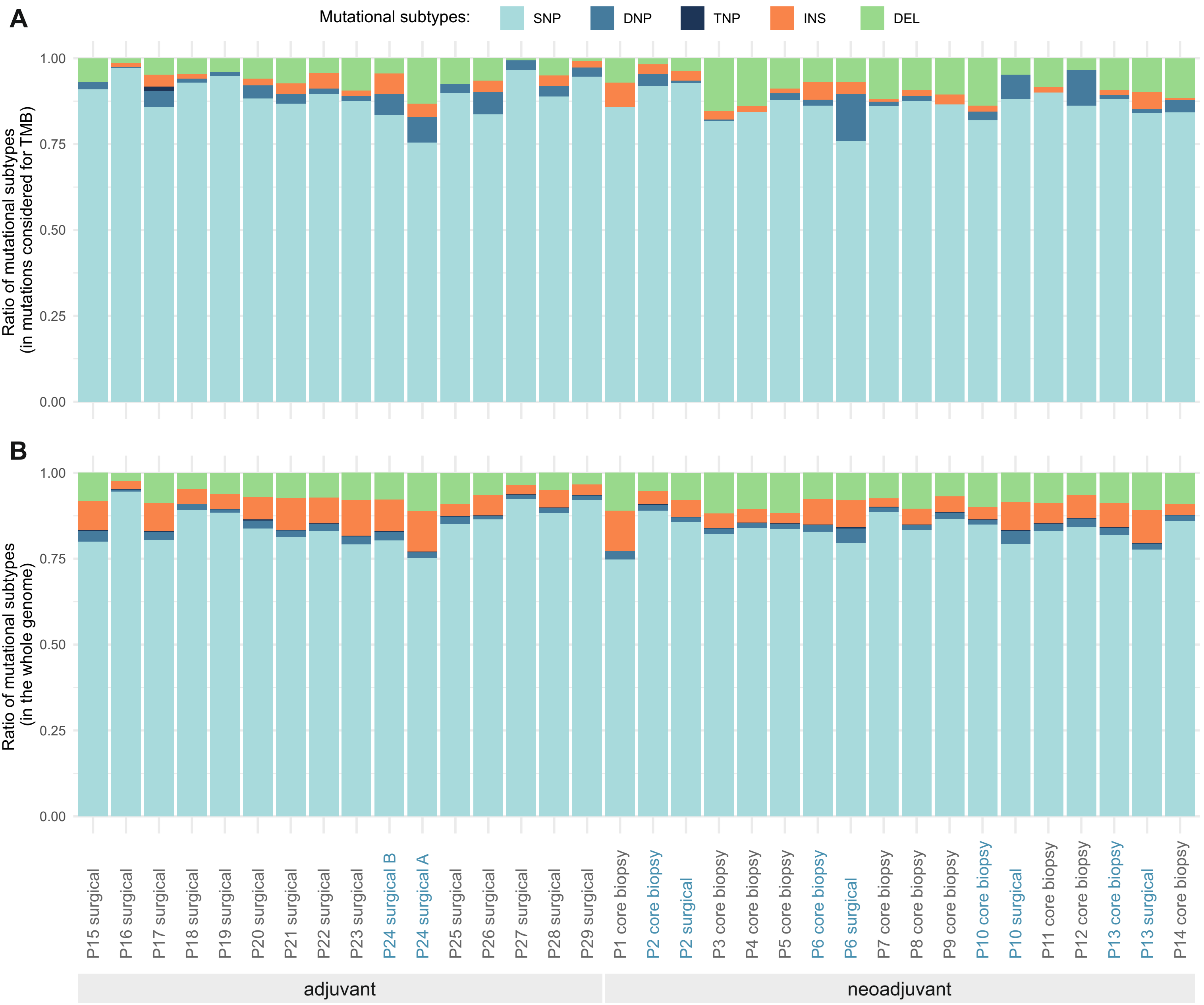

Supplement: Supplementary file 1 [file ijms-24-08553-s001.zip › Supp_Fig_3_new.pdf]
