# Supplementary material for: Genomic Landscape of Normal and Breast Cancer Tissues in a Hungarian Pilot Cohort
Source: Int J Mol Sci. 2023 May 10;24(10):8553. doi: 10.3390/ijms24108553 (PMC10218458; doi:10.3390/ijms24108553)

Supplementary Figure 5. Association between genetic markers of HRD.
Single points on the graphs represent the investigated tumor samples. **A.** Relationship between Single Base Substitution (SBS) Signature 3 contribution and HRD-score. **B.** Relationship between Indel (ID) Signature 6 and 8 total contribution and HRD-score. **C.** Relationship between SBS3 signature and total ID6 and ID8 signature contribution.

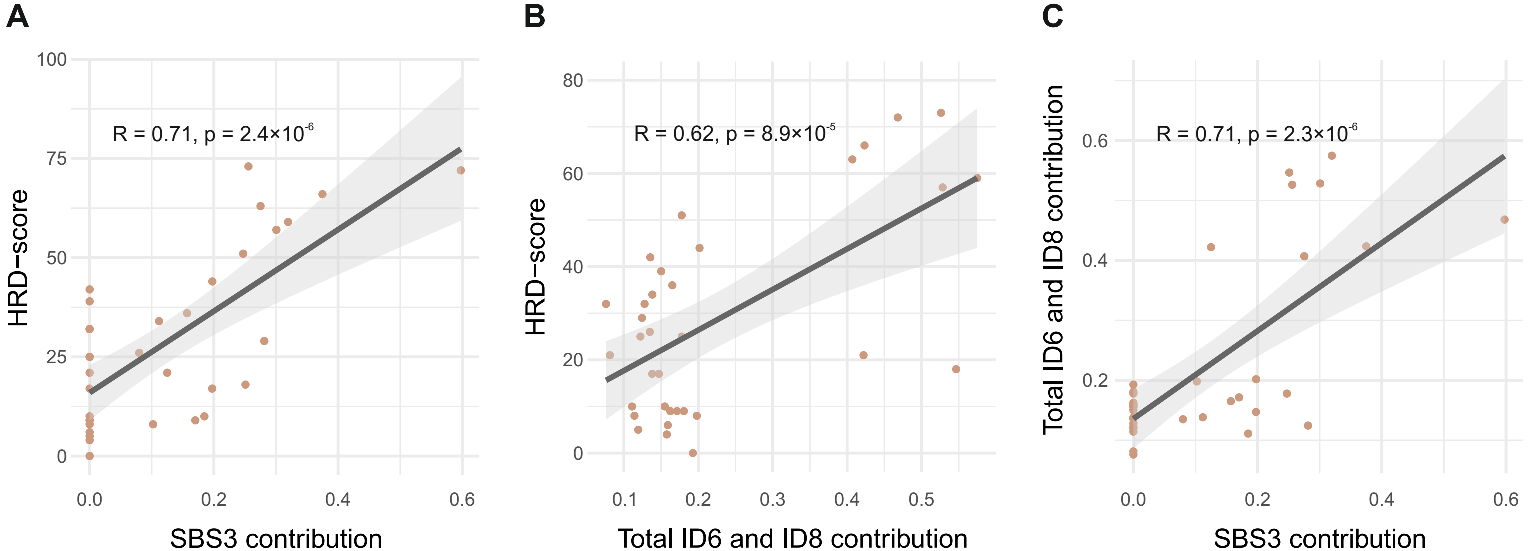

Supplement: Supplementary file 1 [file ijms-24-08553-s001.zip › Supp_Fig_5_new.pdf]
