# Supplementary material for: Genomic Landscape of Normal and Breast Cancer Tissues in a Hungarian Pilot Cohort
Source: Int J Mol Sci. 2023 May 10;24(10):8553. doi: 10.3390/ijms24108553 (PMC10218458; doi:10.3390/ijms24108553)

Supplementary Figure 6. Comparison of mutation detection pipelines. Overlaps of mutations identified from WGS data and mutations detected by the Illumina TruSight Oncology (TSO) 500 assay default pipeline in case of **A.** all germline mutations detected by the TSO panel, **B.** all somatic mutations detected by the TSO panel, **C.** germline exon mutations detected by WGS and **D**. somatic exon mutations detected by WGS.

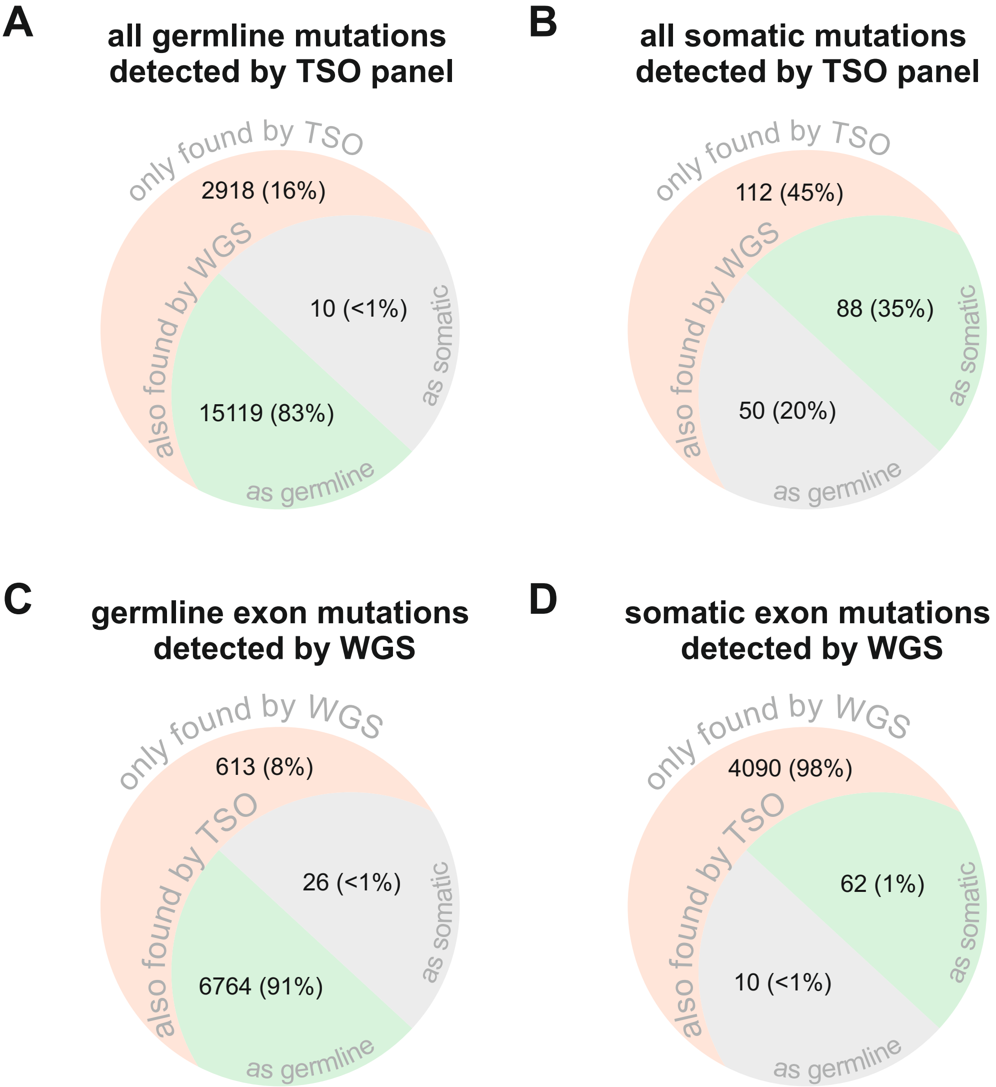

Supplement: Supplementary file 1 [file ijms-24-08553-s001.zip › Supp_Fig_6_new.pdf]
