# Supplementary figures and images for: Genomic Landscape of Normal and Breast Cancer Tissues in a Hungarian Pilot Cohort
Source: Int J Mol Sci. 2023 May 10;24(10):8553. doi: 10.3390/ijms24108553 (PMC10218458; doi:10.3390/ijms24108553)

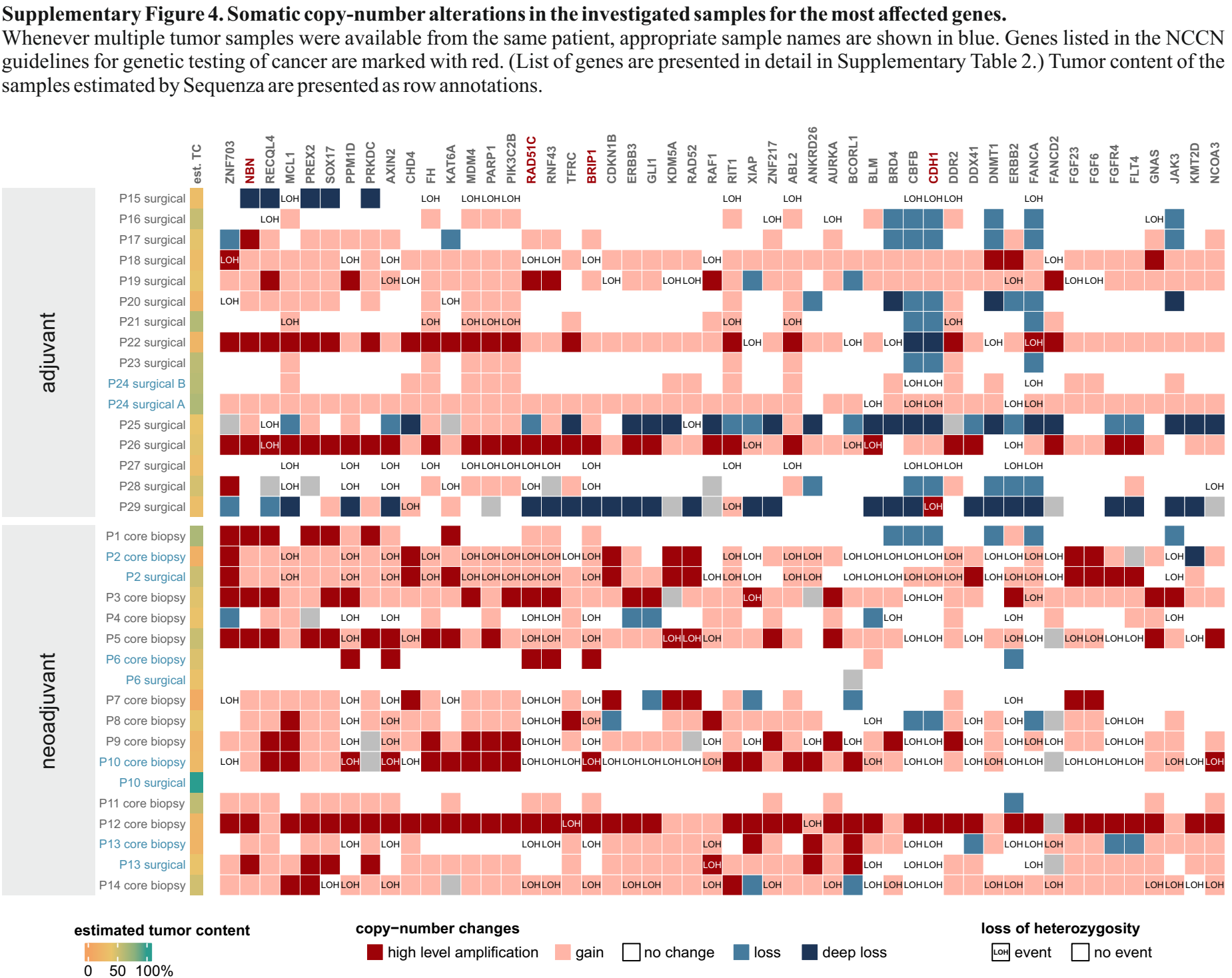

Supplement: Supplementary file 1 [file ijms-24-08553-s001.zip › Supp_Fig_4_new.pdf]
