# Supplementary material for: Genomic Landscape of Normal and Breast Cancer Tissues in a Hungarian Pilot Cohort
Source: Int J Mol Sci. 2023 May 10;24(10):8553. doi: 10.3390/ijms24108553 (PMC10218458; doi:10.3390/ijms24108553)

Supplementary Figure 1. Expanded list of germline mutations.

Germline mutations found in the investigated samples overlapping either the genes listed in the WHO Breast Tumours volume [23] about cancer prevention or the driver genes of lethal metastatic breast cancer established by De Mattos-Arruda et al. [22]. (List of genes are presented in detail in Supplementary Table 2.)

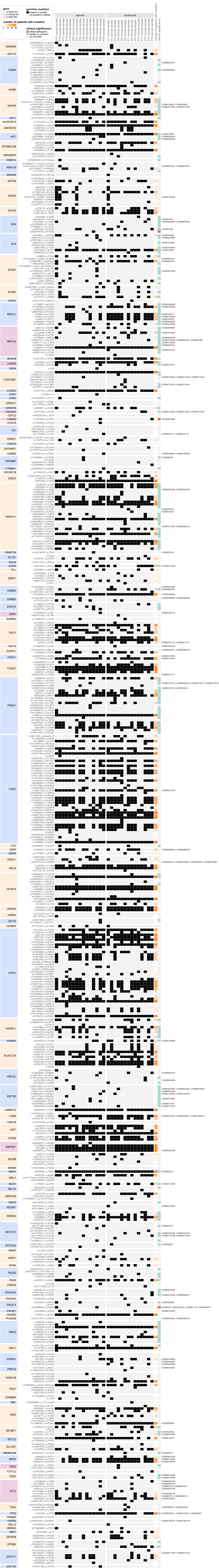

Supplement: Supplementary file 1 [file Supp_Fig_1_new.pdf › Supp_Fig_1_new.pdf]
